# circITGB1 Regulates Adipocyte Proliferation and Differentiation via the miR-23a/ARRB1 Pathway

**DOI:** 10.3390/ijms24031976

**Published:** 2023-01-19

**Authors:** Xiaoyv Yue, Mengdan Fan, Yv Liang, Liying Qiao, Jianhua Liu, Yangyang Pan, Kaijie Yang, Wenzhong Liu

**Affiliations:** College of Animal Science, Shanxi Agricultural University, Jinzhong 030801, China

**Keywords:** circITGB1, miR-23a, ARRB1, ceRNA, adipocytes, proliferation, differentiation

## Abstract

Adipose tissues represent an important energy storage organ in animals and are the largest endocrine organ. It plays an important regulatory role in the pathogenesis of insulin resistance, cardiovascular disease, and metabolic syndrome. Adipose development is a complex biological process involving multiple key genes, signaling pathways, and non-coding RNAs, including microRNAs and circular RNAs. In this study, we characterized circITGB1 and named its host gene *ITGB1*, which is differentially expressed in sheep of different months based on sequencing data. We collated and analyzed the sequencing data to select miRNA-23a with strong binding to *ARRB1*. We found that miRNA-23a regulates the development and differentiation of sheep adipocytes by targeting *ARRB1*. As a competing endogenous RNA, circITGB1 overexpression effectively alleviated the inhibitory effect of miR-23a on *ARRB1*. Conclusively, we provide evidence that circITGB1 regulates the proliferation and differentiation of sheep adipocytes via the miR-23a/ARRB1 pathway. This study provides a scientific basis for further studies on adipose tissue development at the circRNA level.

## 1. Introduction

Obesity has become one of the most serious chronic diseases that endanger human health worldwide owing to the substantial increase in the incidence of obesity caused by insufficient physical activity and unreasonably high energy intake [1,2]. Abnormal lipid metabolism and ectopic deposition are important factors in the occurrence of obesity [3,4]. Lipid metabolism includes lipid synthesis and breakdown, wherein lipid synthesis is related to adipocyte proliferation and differentiation. Sheep are an important source of meat, fur, wool fiber, and dairy products for human consumption [5], and have been used in scientific genetics and disease modeling studies [6,7,8,9].

Circular RNAs (circRNAs) have recently been identified and are competitive endogenous RNA (ceRNA) that bind to miRNA, regulate the expression of target genes, interact with RNA-binding proteins, and encode polypeptides [10,11]. CircRNAs are a class of non-coding RNAs that form covalently bound closed-loop structures with neither 5′ to 3′ polarity nor polyadenylation tails, which are abundant in eukaryotes [12]. CirRNAs are abundant in mammals, are structurally stable, highly conserved, tissue-specific, and have no 3′ end polyA structure and 5′ end cap structure, so they will not be degraded by RNA [13]. Evidence suggests that circRNAs can act as miRNA sponges to regulate gene expression, cell proliferation, differentiation, and apoptosis. Furthermore, circRNAs can bind to AGO2 protein and use their own miRNA response element to adsorb downstream miRNAs to regulate gene expression [14].

MiRNAs are important regulatory factors involved in the proliferation and differentiation of adipocytes [15,16,17,18,19]. MiR-23a negatively regulates the expression of butterfat synthesis-related mRNA [20]. The miR-23a-3p antagomir abrogates the effect of resveratrol on weight loss and glucose and insulin intolerance in gestational diabetic mice [21]. Guo et al. reported that overexpression of miR-23a/b promoted osteogenic differentiation in bone marrow mesenchymal stem cells, whereas inhibition of miR-23a/b increased adipogenic differentiation [22]. An increasing number of studies have shown that the circRNA/miRNA/mRNA regulatory system plays an important role in adipose development [23]. CircFUT10 promotes adipocyte proliferation and inhibits adipocyte differentiation via the let-7c/PPARGCIB pathway [24]. CircSAMD4A controls adipogenesis in obesity via the miR-138-5p/EZH2 axis [25]. Circ-PLXNA1 regulates the differentiation of preadipocytes in Gu ducks via the PLXNA1/miR214/CTNNB1 axis [26]. Therefore, we speculated that circRNAs, as a new regulatory layer, may play an important role in adipocyte differentiation in sheep.

β-Arrestin1 (ARRB1), originally identified as a negative regulator of G-protein-coupled receptor (GPCR) signaling, has been demonstrated to function as a molecular scaffold that regulates cellular function by interacting with other partner proteins. ARRB1 partially represses diet-induced obesity and improves glucose tolerance by interacting with peroxisome proliferator-activated receptor (PPAR)-γ in preadipocytes [27].

Our present study was intended to serve as a starting point for future studies examining the role played by circRNAs in sheep fat development. Our laboratory previously performed high-throughput sequencing on adipose tissue of different growth and development stages of sheep and found that the expression of circITGB1 was significantly different in the two tissues, indicating that circITGB1 may be involved in adipose development, so circITGB1 was selected as the research object. The results demonstrated that circITGB1 binding to miR-23a promoted cell proliferation and inhibited cell differentiation by targeting ARRB1 in adipocytes. This study provides a molecular basis for the study of non-coding RNA regulatory networks in adipocytes and offers an important reference for obesity and obesity-associated metabolic disorders.

## 2. Results

### 2.1. Characterization of circITGB1 in Adipocytes

**Figure 1:**
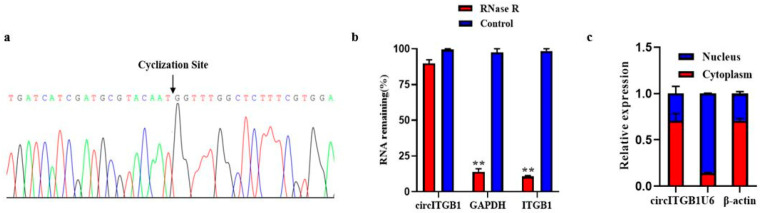
Authenticity identification of CircITGB1 in adipocytes. (**a**) Sanger sequencing of circITGB1 backsplice junctions. (**b**) Relative expression levels of circITGB1 and ITGB1 after RNase R treatment for 30 min, using GAPDH as a reference. (**c**) After nuclear separation, the relative expression of circITGB1 in the nucleus and cytoplasm, using U6 and β-actin as reference. ** *p* < 0.01.

The size and sequence of the amplified PCR products with specific circRNA junctions were confirmed by Sanger sequencing, consistent with the previous sequencing results (Figure 1A). RNA was digested with RNase R exonuclease to further determine the stability of circITGB1. The results showed that circITGB1 was highly resistant to RNase R digestion, while the related linear transcripts (ITGB1 and GAPDH mRNA) were significantly degraded (Figure 1B, *p* < 0.01), proving that circITGB1 has a stable circular structure. Nuclear and cytoplasmic separation experiments showed that circITGB1 mainly exists in the cytoplasm (Figure 1C).

### 2.2. CircITGB1 Promotes Adipocytes Proliferation

**Figure 2:**
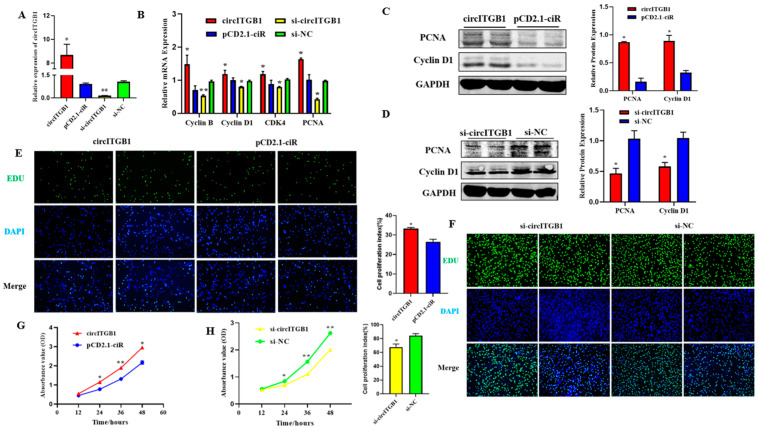
Effect of circITGB1 on the adipocyte proliferation. (**A**) The quantitative PCR (qPCR) results show the expression level of circITGB1 after transfection of adipocytes with pCD2.1-circITGB1 and si-circITGB1. (**B**) After transfection of adipocytes with pCD2.1-circITGB1 and si-circITGB1, the mRNA expressions of cyclin B, cyclin D1, CDK4, and PCNA were detected using qPCR. (**C**) After transfection of adipocytes with pCD2.1-circITGB1, the protein expressions of cyclin D1 and PCNA were detected by Western blot. (**D**) After transfection of adipocytes with si-circITGB1, the protein expressions of cyclin D1 and PCNA were detected by Western blot. (**E**,**F**) Cell proliferation analysis using the EdU assay. (**G**,**H**) Cell proliferation analysis using the Cell Counting Kit-8 (CKK-8) assay. Scale bar = 100 mm.* *p* < 0.05, ** *p* < 0.01.

To determine the effect of circITGB1 on adipocyte proliferation, transfection of the circITGB1 overexpression plasmid pCD2.1-circITGB1 and the interference plasmid si-circITGB1 into sheep adipocytes significantly increased and decreased the expression level of circITGB1 (Figure 2A, *p* < 0.05). Consistently, pCD2.1-cir-cITGB1 promoted the expression of cyclin B, cyclin D1, cyclin-dependent kinase 4 (CDK4), and proliferating cell nuclear antigen (PCNA) at the mRNA level, and si-circITGB1 is just the opposite (Figure 2B, *p* < 0.05). Cyclin D1 and PCNA protein expression levels were consistent with the gene expression results (Figure 2C, *p* < 0.05). As expected, the expression of Cyclin D1 and PCNA was significantly decreased when circITGB1 was inhibited, indicating that circITGB1 promotes adipocyte proliferation (Figure 2D, *p* < 0.05). EdU staining analysis showed that circITGB1 overexpression increased the number of EdU-positive cells, indicating that it could promote adipocyte proliferation, and the results of the si-circITGB1 group were just the opposite (Figure 2E,F, *p* < 0.05). The results of the Cell Counting Kit-8 (CCK-8) assay showed that the regulation of circITGB1 enhanced the viability of adipocytes (Figure 2G,H, *p* < 0.05).

### 2.3. CircITGB1 Inhibits Adipocytes Differentiation

**Figure 3:**
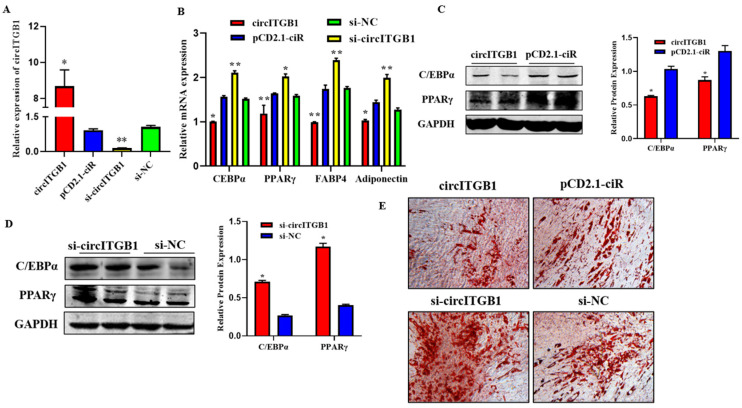
Effect of circITGB1 on adipocyte differentiation. (**A**) The quantitative PCR (qPCR) results show the expression level of circITGB1 after transfection of adipocytes with pCD2.1-circITGB1 and si-circITGB1. (**B**) The mRNA expression levels of C/EBPa, PPARγ, FABP4, and adiponectin in adipocytes by qPCR. (**C**,**D**) The protein expression levels of PPARγ and C/EBPa in adipocytes were indicated by Western blot. (**E**) Oil red O staining revealed that circITGB1 inhibited lipid droplet formation. * *p* < 0.05, ** *p* < 0.01.

To verify the impact of circITGB1 on adipocyte differentiation, plasmid pCD2.1-circITGB1 and si-circITGB1 were transfected into adipocytes, respectively. The expression level of circITGB1 in the pCD2.1-circITGB1 group was significantly higher than that in the pCD2.1-ciR group and the si-circITGB1 group was significantly lower than that in the si-NC group (Figure 3A; *p* < 0.01). After circITGB1 overexpression, the mRNA expression levels of the established adipocyte markers, CCAAT/enhancer-binding protein α (C/EBPα), peroxisome proliferator-activated receptor γ (PPARγ), fatty acid binding protein 4 (FABP4), and adiponectin were significantly decreased, while the results in the si-circITGB1 group were the opposite (Figure 3B; *p* < 0.01). The protein levels of PPARγ and C/EBPα were significantly decreased in the pCD2.1-circITGB1 group and increased in the si-circITGB1 group (Figure 3C,D, *p* < 0.05). Adipocyte differentiation was induced for eight days and analyzed using Oil Red O staining. The results showed that circITGB1 in adipocytes produced fewer lipid droplets than the pCD2.1-ciR group (Figure 3E).

### 2.4. MiR-23a Inhibits Adipocytes Proliferation

**Figure 4:**
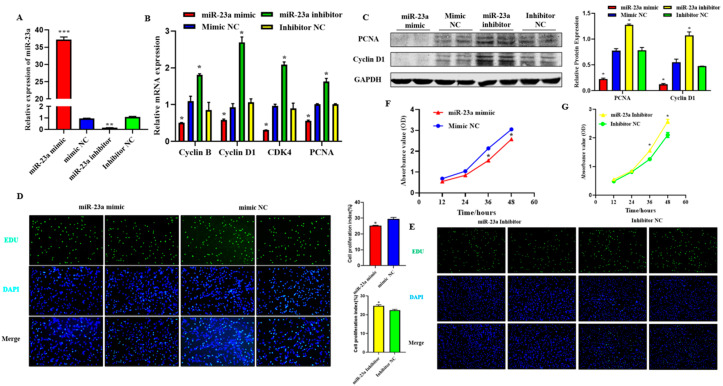
Effect of miR-23a on adipocyte proliferation. (**A**) Expression levels of miR-23a in adipocytes by qPCR. (**B**) Expression levels of cyclin B, cyclin D1, CDK4, and PCNA in adipocyte by qPCR. (**C**) Expression levels of Cyclin D1 and PCNA by Western blot. (**D**,**E**) Cell proliferation analysis using the EdU assay. (**F**,**G**) Cell proliferation analysis using Cell Counting Kit 8 (CCK-8) assay. * *p* < 0.05, ** *p* < 0.01, *** *p* < 0.001.

To explore the function of miR-23a on adipocyte proliferation, miR-23a mimic and inhibitor were transfected into adipocytes at 80 nM to detect whether an overexpression or inhibitory effect was achieved. The results showed that the two oligonucleotides of miR-23a had the expected effect compared with the mimic NC group and inhibitor NC group, respectively, and could be used in consecutive experiments (Figure 4A, *p* < 0.01). qPCR and Western blotting results showed that after overexpression of miR-23a, the mRNA expression levels of proliferation marker genes cyclin B, cyclin D1, CDK4, and PCNA were significantly lower than those in the control group, and the protein expression levels of PCNA and Cyclin D1 were also significantly decreased. As expected, the expression of both proliferation marker genes and proteins was significantly increased when miR-23a was inhibited, indicating that miR-23a inhibits adipocyte proliferation (Figure 4B,C, *p* < 0.05). EdU staining revealed that the number of new adipocytes in the mimic group was significantly lower than that in the control group (Figure 4D, *p* < 0.05), while the results in the inhibitor group were the opposite (Figure 4E, *p* < 0.05), indicating that miR-23a could inhibit the formation of new adipocytes. Compared with the control group, the absorbance of the adipocytes in the mimic group decreased significantly at 36 h and 48 h (Figure 4F, *p* < 0.05), while the absorbance of the inhibitor group decreased significantly at 36 h and 48 h (Figure 4G, *p* < 0.05). This indicated that regulation of miR-23a could suppress the viability of adipocytes.

### 2.5. MiR-23a Promotes Adipocytes Differentiation

**Figure 5:**
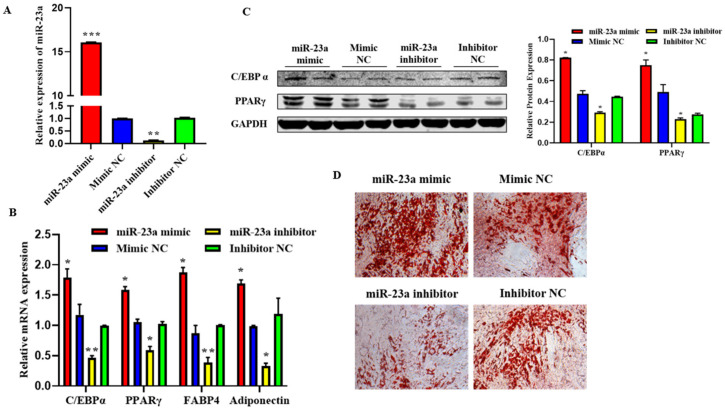
Effect of miR-23a on the adipocyte differentiation. (**A**) Expression levels of miR-23a in adipocytes by quantitative PCR (qPCR). (**B**) Expression levels of C/EBPa, PPARγ, FABP4, and adiponectin in adipocytes by qPCR. (**C**) Expression levels of C/EBPa and PPARγ by Western blot. (**D**) Oil red O staining. * *p* < 0.05, ** *p* < 0.01, *** *p* < 0.001.

To investigate the possible role of miR-23a in adipocyte differentiation, we transfected 80 nM miR-23a mimic and inhibitor into adipocytes. The qPCR results showed that the two oligonucleotides of miR-23a were significantly different from the NC group, indicating that the transfection of the miR-23a mimic and inhibitor was successful and could be used in subsequent experiments (Figure 5A, *p* < 0.01). The qPCR and Western blotting results showed that after overexpression of miR-23a, the mRNA expression levels of the differentiation marker genes C/EBPα, PPARγ, FABP4, and adiponectin were significantly higher than those in the control group, and the protein expression levels of C/EBPα and PPARγ were significantly increased. When miR-23a was inhibited, the expression levels of marker genes and proteins significantly decreased (Figure 5B,C, *p* < 0.05). This indicates that miR-23a promotes adipocyte differentiation. After transfection with miR-23a mimic/mimic NC and miR-23a inhibitor/inhibitor NC for eight days, Oil Red O staining was performed in adipocytes. The results showed that miR-23a plays an important role in adipocyte differentiation, contributing to adipose deposition (Figure 5D).

### 2.6. ARRB1 Is a Target of miR-23a

**Figure 6:**
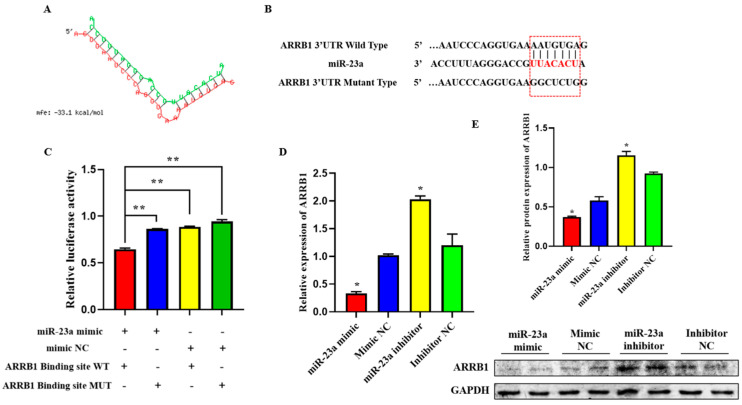
*ARRB1* is a Target of miR-23a. (**A**) Predicted binding site between miR-23a and *ARRB1*. (**B**) The predicted binding site and mutated site of miR-23a in the 3′UTR of ARRB1. (**C**) The pmirGLO-ARRB1-WT and pmirGLO-ARRB1-MUT plasmids were co-transfected with mi-23a mimics or NC into 293 T cells. Luciferase activity was determined 48 h post-transfection. (**D**,**E**) miR-23a repressed ARRB1 expression at both the mRNA and protein levels in adipocytes. * *p* < 0.05, ** *p* < 0.01.

To elucidate the mechanisms whereby miR-23a regulates adipocyte proliferation and differentiation, we predicted potential miR-23a targets using RNAhybrid and found that the 3′-UTRs of *ARRB1* contain conserved target sites for miR-23a (Figure 6A). To investigate whether miR-23a targets the *ARRB1* 3′-UTR, wild-type and mutated sequences containing the binding sites were inserted into the pmirGLO vector to construct a pmirGLO dual-luciferase miRNA target expression vector (Figure 6B). After co-transfection of vectors and miR-23a mimics (or mimic NC) into 293T cells, the relative luminescence activity of the group with wild-type reporter and mimic was significantly lower compared with the control groups, which were transfected with mutated reporters and mimic, wild-type reporter, and mimic NC, respectively (Figure 6C, *p* < 0.01), indicating that miR-23a and *ARRB1* 3′-UTR have a targeted relationship. Consistently, we found that miR-23a repressed *ARRB1* expression at both the mRNA and protein level in adipocytes (Figure 6D,E, *p* < 0.05). In conclusion, these results suggest that *ARRB1* is a target of miR-23a.

### 2.7. CircITGB1 Act as a ceRNA for miR-23a

**Figure 7:**
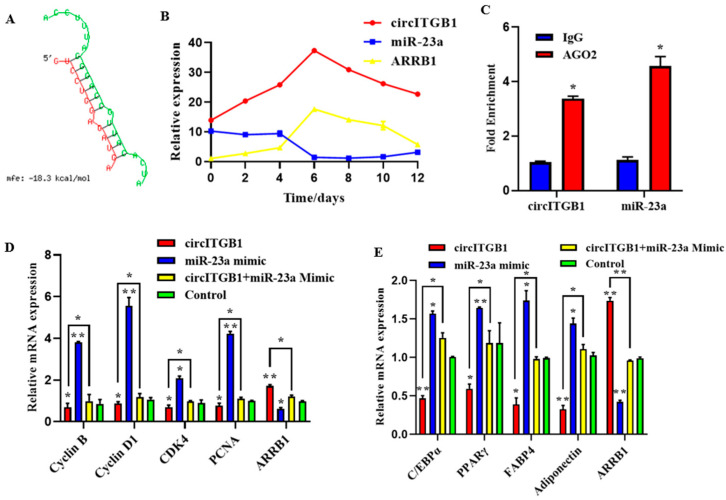
CircITGB1 act as a ceRNA for miR-23a. (**A**) Predicted binding site between circITGB1 and miR-23a. (**B**) Expression trends of circITGB1, miR-23a, and *ARRB1* during adipocyte differentiation (**C**) The results of the RNA-binding protein immunoprecipitation experiment were compared with IgG. (**D**) Expression levels of cyclin B, cyclin D1, CDK4, PCNA, and ARRB1 in adipocytes by qPCR. (**E**) Expression levels of C/EBPα, PPARγ, FABP4, adiponectin, and ARRB1 in adipocyte by qPCR. * *p* < 0.05, ** *p* < 0.01.

CircRNAs can participate in the post-transcriptional regulation of genes. The binding sites of circITGB1 and miR-23a were predicted using the RNAhybrid online software. We found potential binding sites for circITGB1 to the seed region of miR-23a (Figure 7A), consistent with the sequencing data. The qPCR results showed that during adipocyte differentiation, the expression of miR-23a reached its lowest value at day six, whereas circITGB1 and ARRB1 mRNA reached their highest value (Figure 7B). The three expression trends indicated that there was a negative correlation between the expression of circITGB1 and miR-23a and the expression of miR-23a and *ARRB1*. Next, an RNA-binding protein immunoprecipitation (RIP) assay revealed that both circITGB1 and miR-23a could interact with the AGO2 protein, indicating a potential interaction between circITGB1 and miR-23a (Figure 7B, *p* < 0.05).

**Figure 8:**
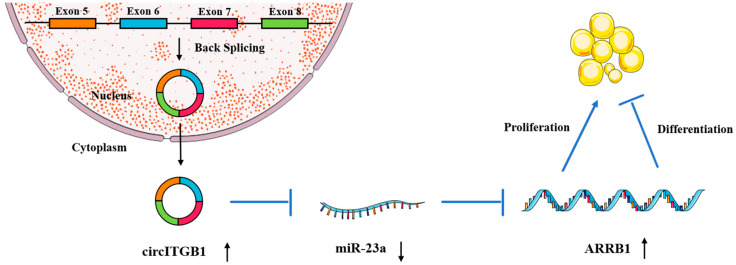
Hypothetical model for the interaction of circITGB1 with miR-23a and its effects on *ARRB1* and adipocyte proliferation and differentiation. CircITGB1 enhances *ARRB1* expression by acting as a miRNA sponge. CircITGB1 enhances the proliferation of adipocytes and inhibits their ability to differentiate, thereby inhibiting the progression of lipid droplet formation.

To determine whether the regulatory effects of circITGB1 on adipocytes were dependent on miR-23a, the miR-23a mimic and pCD2.1-circITGB1 were separately co-transfected into adipocytes. MiR-23a overexpression inhibited the expression of the cell proliferation marker genes cyclin B, cyclin D1, CDK4, and PCNA at the mRNA level, but co-transfection with circITGB1 reversed the inhibitory effect of miR-23a on adipocytes (Figure 7C, *p* < 0.05). Similarly, miR-23a overexpression significantly increased the expression of the differentiation marker genes C/EBPα, PPARγ, and FABP4 compared with the control group, and this effect was suppressed after co-transfection with circITGB1 (Figure 7D, *p* < 0.05). Furthermore, circITGB1 introduction relieved the effect of miR-23a on *ARRB1* (Figure 7D, *p* < 0.05). Taken together, these results demonstrated that circITGB1 adipocytes promote proliferation and inhibit differentiation by competitively binding to miR-23a. As shown in Figure 8, circITGB1 acts as an miR-23a sponge to promote *ARRB1* expression, thereby regulating adipocyte development. 

## 3. Discussion

With the improvement in living standards, people’s demand for high-quality meat is becoming increasingly urgent, and high-quality meat mainly depends on two factors: muscle development and fat deposition. To better control body fat deposition, livestock can be enriched with fat through technical means or genetic improvement interventions. Exploring the regulatory mechanism of fat deposition helps control the lipid deposition ability of livestock and can also improve the lean meat rate and meat quality of livestock. Simultaneously, fat can improve cold tolerance in animals, especially in newborn animals. This can improve the survival rate, reduce production costs, and improve economic benefits. CircRNAs are abundantly expressed in mammals and are tissue-specific [28], with a generation efficiency that is sometimes higher than that of linear homologous mRNA [29]. The circular closed structure of circRNA is resistant to exonuclease, can resist the digestion of RNase R enzyme, and has high stability [30,31]. Presently, research on circRNAs in sheep mainly focuses on muscle development [32] and reproductive performance [33,34,35]. There are relatively few studies on the mechanism of circRNAs in the proliferation and differentiation of adipocytes. In recent years, increasing evidence has shown that abnormal expression of non-coding RNA (ncRNAs) is related to the occurrence and development of diseases [36,37,38,39]. In this study, sheep adipocytes were used as the research object to explore the mechanism of circITGB1 on adipose deposition in animals to improve the lean meat rate of livestock and poultry and improve meat quality. Here, we selected circITGB1, a novel circRNA, as the research object based on previous laboratory sequencing data. Using PCR amplification, nucleoplasmic localization, and RNase R experiments, circITGB1 was generated from ITGB1 exons by back-splicing, which is mainly located in the cytoplasm. Regarding the regulation of the adipocyte proliferation process, the expression levels of the proliferation marker genes PCNA, cyclin B, cyclin D1, and CDK4 are often used to reflect the proliferation level of adipocytes [40], and the adipogenic differentiation marker genes PPARγ, C/EBPα, adiponectin, and FABP4 are used to reflect the degree of adipocyte differentiation [41]. In this study, the presence of circITGB1 promoted the expression of proliferation marker genes cyclin B, cyclin D1, CDK4, and PCNA in adipocytes while inhibiting the expression of differentiation marker genes PPARγ, C/EBPα, FABP4, and adiponectin.

Most studies have shown that miRNAs play inhibitory roles at both transcriptional and translational levels by binding to their target genes [42,43]. Because each miRNA can complement many mRNAs, they have the potential to regulate multiple genes [44]. MiRNAs are involved in the post-transcriptional regulation of gene expression in almost all preadipocyte events, including cell proliferation, differentiation, and apoptosis [45,46,47,48,49]. In this study, by transfecting sheep adipocytes with miRNA, it was found that miR-23a upregulated the mRNA and protein expression of proliferation marker genes and downregulated the mRNA and protein expression of adipogenic differentiation marker gene mRNA. This indicates that miR-23a inhibits adipocyte proliferation and promotes adipocyte differentiation. The results of CCK-8, EdU detection, and Oil Red O staining further confirmed this conclusion. The study found that the overexpression of miR-23a-5p enhanced adipogenesis through IGF2 targeting, which is consistent with the results of this experiment [50].

Using bioinformatical prediction, *ARRB1* was identified as the target of miR-23a. The results of this study revealed that miR-23a regulates the *ARRB1* expression at both the mRNA and protein level. During the differentiation process, the expression trends of miR-23a and ARRB1 were opposite, which may reveal a negative regulatory relationship between the two to a certain extent. In this study, the ARRB1-3′UTR wild-type and mutant sequences containing the miR-23a binding site were constructed into a fluorescein reporter vector and transfected into 293T cells together with the miR-23a overexpressing mimic fragment. The results showed that the fluorescence activity of transfected wild-type ARRB1-3′UTR and miR-23a mimic decreased, indicating that there is a targeting relationship between ARRB1-3′UTR and miR-23. Studies have shown that *ARRB1* inhibits PPARγ-mediated transcriptional activity [51], which may provide a basis for explaining why miR-23a can promote adipose differentiation via *ARRB1* inhibition.

Circular RNAs can bind to specific miRNAs as competitive endogenous RNAs, thereby inhibiting the effects of miRNAs on their target mRNAs. Therefore, the next question was whether circITB1 relieves the inhibitory effect of miR-23a on downstream ARRB1 through competitive binding. Bioinformatic analysis revealed that circITGB1 has a binding site with the seed region of miR-23a. The competing endogenous RNA (ceRNA) hypothesis states that circRNAs can regulate gene expression by binding to the AGO2 protein and using their own miRNA response elements (MRE) to adsorb downstream miRNAs. To further determine the relationship between circITGB1 and miR-23a, we performed an RNA immunoprecipitation assay. The results showed that both circITB1 and miR-23a could interact with the AGO2 protein, suggesting a potential interaction between circITB1 and miR-23a. RIP, quantitative real-time PCR, and Western blotting assays indicated that circITGB1 directly binds to miR-23a. Under miR-23a overexpression, circITGB1 overexpression in turn restored the proliferation and differentiation abilities of adipocytes. In conclusion, circITGB1 acts as a sponge for miR-23a, which can relieve the inhibitory effect on the downstream target gene ARRB1, thereby promoting proliferation and inhibiting the differentiation of adipocytes.

The adipose tissue is an important endocrine organ. Adipose tissue can store energy, maintain a constant body temperature, buffer external pressure, and promote the absorption of fat-soluble vitamins, thereby providing the body with essential fatty acids. Appropriate fat content is an important indicator for evaluating meat quality. Adipose tissue is also closely related to certain physiological and pathological processes such as glucose and fat metabolism, obesity, diabetes, fatty liver, hyperlipidemia, and breast cancer. Since circITGB1 plays a role in adipocyte proliferation and differentiation, circITGB1 may have a relationship with obesity and other related diseases, such as diabetes, which requires further elucidation.

## 4. Materials and Methods

### 4.1. Sample Collection

The adipose tissue used in this study was taken from Guanglin big-tail sheep at five months of age. Animal experiments presented in this study were performed in accordance with the Guidelines for the Care and Use of Laboratory Animals prepared by the Institutional Animal Care and Use Committee of Shanxi Agricultural University, Taigu, Shanxi, China.

### 4.2. Cell Isolation and Culture

Adipocytes were obtained from the subcutaneous adipose tissue of 5-month-old sheep according to the previously published method with minor revision [52]. Following death, subcutaneous adipose tissue was collected, placed in sterile pre-cooled phosphate-buffered saline (PBS), and brought back to the laboratory for cell isolation and culture. The adipose tissue was finely minced by surgery and digested with 2 mg/mL collagenase type II (C8150, Solarbio, Beijing, China) for 1 h at 37 °C. Digestion was terminated using complete medium containing 89% Dulbecco’s Modified Eagle’s medium (DMEM; C11995500BT, Gibco, USA), 10% fetal bovine serum (FBS; 04-001-1A, Biological Industries, Israel), and 1% penicillin–streptomycin–neomycin (PSN) antibiotic. The mixture was centrifuged at 500× *g* for 8 min at room temperature. The cell pellet was resuspended in fresh complete medium. The suspension was then filtered using 75 and 40 micro nylon mesh to remove incompletely digested tissue debris. Cells were cultured in complete medium at 37 °C and 5% CO_2_. The culture medium was changed every 24 h. When the confluency of adipocytes reached 80%, the medium was replaced with differentiation medium containing 89% DMEM, 10% FBS, 1% PSN antibiotic, 250 μmol·L^−1^ 3-isobutyl-1-methylxanthine (IBMX; I8450, Solarbio, Beijing, China), 500 μmol·L^−1^ dexamethasone (ID0170, Solarbio, Beijing, China) and 8 μmol·L^−1^ insulin (I8040, Solarbio, Beijing, China).

### 4.3. Plasmids Construction

The expression plasmid of circITTGB1 was constructed by inserting full-length circITGB1 (578 bp) into pCD2.1-ciR (Wuhan GeneCreate Bioengineering Co., Ltd., Wuhan, China) between KpnI and BamHI restriction sites. To knock down circITGB1, siRNAs targeting the back-splice junction site of circITGB1 and an siRNA-NC were synthesized by Ruibo (Guangzhou, China) Oar-miR-23a mimic, mimic negative control (mimic NC), 2′-O-methyl antisense oligonucleotide against miR-23a (miR-23a inhibitor), inhibitor negative control (inhibitor) NC), pmirGLO-ARRB1-WT, and pmirGLO-ARRB1-MUT were purchased from Sangon Biotech Co., Ltd. (Shanghai, China).

### 4.4. Cell Transfection

All transient transfections were performed using Lipofectamine 3000 reagent (Invitrogen, Carlsbad, CA, USA), according to the manufacturer’s instructions.

### 4.5. RNA Exaction, cDNA Synthesis, and Quantitative Real-Time PCR

All RNA was extracted using TRIzol reagent (TaKaRa, Otsu, Japan) according to the manufacturer’s instructions. RNA quantity and purity were assessed using an RNA 6000 Nano LabChip Kit and Bioanalyzer 2100 (Agilent) [53]. Total RNAs were reverse-transcribed into cDNA using random primers, according to the manufacturer’s instructions (Roche, Penzberg, Germany).

### 4.6. Primer

Primers used in this study were synthesized by Sangon Biotech (Shanghai, China). The primers listed in Table 1 were designed for the Oligo7.0 software (Premier Bio-soft International, Palo Alto, CA, USA).

### 4.7. Validation of circITGB1

Using the cDNA of sheep adipocytes as a template, the target fragment containing the interface was amplified by PCR. After the target fragment was amplified, agarose gel electrophoresis was performed, and the corresponding PCR products were sequenced by Sangon Biotech (Shanghai, China). Total RNAs (5 μg) were digested with RNase R (3U·μg^−1^) (GeneSeed, Guangzhou, China) or enzyme-free water as a control at 37 °C for 30 min, and 70 °C for 10 min to remove linear RNAs and enrich circular RNAs according to the manufacturer’s protocols. RNA was re-extracted, and reverse-transcribed into cDNA, and qPCR was used to detect the mRNA expression of sheep circITGB1, ITGB, and GAPDH. To analyze the effect of circITGB1 depending on cellular localization, we used NE-PER nuclear and cytoplasmic extraction reagents (Thermo Scientific) to extract the nuclear and cytoplasmic fractions according to the instructions, then stored them at −80 °C for subsequent use. Nuclear and cytoplasmic RNA was extracted using Trizol reagent, and the expression of circITGB1, U6, and β-actin mRNA in the nucleus and cytoplasm was detected by qPCR.

### 4.8. EdU and CCK-8 Analysis

Adipocyte proliferation was evaluated using the Cell-LightTM EdU DNA Cell Proliferation Kit (C10310-1; RiboBio, Guangzhou, China) according to the manufacturer’s instructions, and adipocytes in the logarithmic growth phase were evenly seeded into 96-well plates. When the confluency of the cultured cells reached 70%, cells were transfected with six replicates in each group. A 96-well plate was removed 12, 24, 36, and 48 h after transfection. Ten microliters of CCK-8 solution were added to each well, and after incubation for 2 h, the absorbance value at 450 nm was detected using a multifunction microplate reader, and the cell proliferation curve was drawn.

### 4.9. Western Blotting

Total proteins were extracted using radioimmunoprecipitation assay buffer (RIPA) with 1% PMSF (Solarbio, Beijing, China) after transfection for 48 h or induction for six days after transfection. The extracted protein was mixed with protein loading buffer (denaturation) at a ratio of 4:1 and denatured at 100 °C for 10 min. Then, protein was subjected to 10% polyacrylamide gel electrophoresis at 80 V for 30 min and 120 V for 90 min and transferred to a 150 V ice bath for 40 min. The skim milk powder was blocked for 1 h, and the primary antibody was incubated overnight. The antibodies were then rinsed with PBS-Tween three times for 5 min each. The fluorescent secondary antibody was incubated in the dark for 2 h and washed with PBST three times for 5 min each. The primary antibodies used were against cyclin D1 (1:1000, bs-20596R, Bioss), proliferating cell nuclear antigen (PCNA; 1:1000, bs-0754R), peroxisome proliferator-activated receptor γ (PPARγ; 1:1000, bs-4590R), CCAAT/enhancer-binding protein alpha (C/EBPα; 1:1000, bs-1630R), phospho-beta-arrestin (ARRB1; 1:1000, bs-3048R), and GAPDH (1:5000, bs-2188R). The secondary antibody used was goat anti-rabbit IgG H&L/AP (1:5000, bs-0295G-AP). Finally, we present the results of Western blotting using Image Studio Lite ver. 5.2 (LI-COR Inc., Lincoln, NE, USA) and quantified with the ImageJ program (Bio-Rad, Hercules, CA, USA).

### 4.10. Oil Red Staining

The differentiated and mature cells were removed from the cell culture incubator, washed three times with PBS, mixed with 4% paraformaldehyde in PBS, fixed at room temperature for 30 min, and then discarded. PBS was added twice for 5 min each. Add 60% isopropanol in PBS, and permeabilize for 30 s. This step makes it easier for the Oil Red O stain to enter the cells. Oil Red staining solution was added to the wells of the culture plate, incubated at room temperature for 1 h, washed three times with PBS, and micrographed.

### 4.11. Biding Relationship Prediction

The relationship between miR-23a, ARRB1, circRNA, and miR-23a was predicted using the RNAhybrid website (https://bibiserv.cebitec.unibiele.org.de/rnahybrid) [54], accessed on 1 January 2023.

### 4.12. Dual-Luciferase Reporter Assay

HEK-293T cells were seeded in a 12-well culture plate. When the cells reached 70% confluence, they were transfected with Lipofectamine 3000. The test was divided into three groups with four repetitions per group. Co-transfected pmirGLO-ARRB1-3′-UTR-wt and miR-23a mimic (wild-type group), pmirGLO-ARRB1-3′-UTR-mut and miR-23a mimic (mutation group), and pmirGLO-ARRB1-3′-UTR-wt and miR-23a mimic NC (negative control group). After 48 h of transfection, fluorescence activity was detected using the Dual-Luciferase Reporter Assay System.

### 4.13. RIP Assay

The EZMagna RIP RNA-binding protein immunoprecipitation kit (Millipore, Billerica, MA, USA) was used for RIP experiments. First, preadipocyte proteins were extracted. To prepare the magnetic beads for immunoprecipitation, 50 μL of A/G magnetic bead suspension was added to the protein sample, 500 μL of RIP washing solution was added, and the mixture was stirred gently. The centrifuge tube was placed on a magnetic stand, the magnetic beads were gathered on one side of the centrifuge tube wall, and the supernatant was removed. Then, 500 μL of the RIP washing solution was added to the centrifuge tube, shaken gently, washed with magnetic beads, and the supernatant was removed. Then, 100 μL of RIP washing solution was added to the centrifuge tube, Ago2 antibody (bs-12450R; Bioss) was added, and IgG antibody (bs-0297P; Bioss) was added to the negative control group and incubated at room temperature for 30 min. The supernatant was removed, magnetic beads were washed again, and the supernatant was aspirated. Next, 500 μL of the RIP wash solution was added to the centrifuge tube, vortexed briefly, transferred the centrifuge tube to ice, and the supernatant was removed. The obtained RNA-target protein complex was subjected to RNA purification followed by qPCR analysis to further analyze the expression of circITGB1 and miR-23a.

### 4.14. Statistical Analysis

Three samples were used for each experiment, and each sample was tested thrice. The qPCR results used GAPDH and U6 as internal reference genes, and the obtained data were analyzed for significance using the £ test and plotted using GraphPad Prism 7.0. Results were expressed as the mean ± standard error of the mean (SEM), and statistical significance was set at *p* < 0.05.

## Figures and Tables

**Table 1:** Primer sequences for quantitative (qPCR).

Name	Sequence (5′-3′)	Length (bp)
Cyclin B	CGTACTCCGTCTCCAGCC	261
	AGCCAGTCAATCAGGATGGC	
Cyclin D1	GATGCCAACCTCCTCAACGA	211
	GGAAGCGGTCCAGGTAGTTC	
PCNA	ATCAGCTCAAGTGGCGTGAA	213
	TGCCAAGGTGTCCGCATTAT	
CDK4	CCAATGTTGTCCGGCTGATG	150
	CCTTGATCGTTTCGGCTGG	
C/EBPα	TCCGTGGACAAGAACAGCAA	137
	TCATTGTCACTGGTCAGCTCC	
PPARγ	CGTACTCCGTCTCCAGCC	234
	AGCCAGTCAATCAGGATGGC	
FABP4	AAACTGGGATGGGAAATCAACC	261
	TGCTCTCTCGTAAACTCTGGTAGC	
Adiponectin	ATCCCCGGGCTGTACTACTT	129
	CTGGTCCACGTTCTGGTTCT	
circITGB1	TGCGTACAATGGTTTGGCTC	185
	ATGCGCTGCTTACCAACAAG	
ITGB1	GTGGATCCCTTGTCCCACTG	158
	ACCACACCTGCTACAATCGG	
GAPDH	ACAGTCAAGGCAGAGAACGG	98
	CCAGCATCACCCCACTTGAT	
U6	AGCCTTCAAATCACTGGCTACA	197
	AGTACCTGCTTACCCATACCT	
miR-23a	GGCGATCACATTGCCAGGGATTTCCA	

## Data Availability

Data presented in this study are available in the article.
